# Trends in incidence and mortality of tuberculosis in India over past three decades: a joinpoint and age–period–cohort analysis

**DOI:** 10.1186/s12890-021-01740-y

**Published:** 2021-11-16

**Authors:** Deepak Dhamnetiya, Priyanka Patel, Ravi Prakash Jha, Neha Shri, Mayank Singh, Krittika Bhattacharyya

**Affiliations:** 1 Department of Community Medicine, Dr. Baba Saheb Ambedkar Medical College and Hospital, Delhi, 110085 India; 2 Department of Development Studies, International Institute for Population Sciences (IIPS), Mumbai, 400088 India; 3 International Institute for Population Sciences (IIPS), Mumbai, 400088 India; 4 Department of Fertility Studies, International Institute for Population Sciences (IIPS), Mumbai, 400088 India; 5 Department of Statistics, University of Calcutta, Kolkata, 700019 India

**Keywords:** Tuberculosis, Incidence, Mortality, Joinpoint regression analysis, India, GBD

## Abstract

**Background:**

Tuberculosis, as a communicable disease, is an ongoing global epidemic that accounts for high burden of global mortality and morbidity. Globally, with an estimated 10 million new cases and around 1.4 million deaths, TB has emerged as one of the top 10 causes of morbidity and mortality in 2019. Worst hit 8 countries account for two thirds of the new TB cases in 2019, with India leading the count. Despite India's engagement in various TB control activities since its first recognition through the resolution passed in the All-India Sanitary Conference in 1912 and launch of first National Tuberculosis Control Programme in 1962, it has remained a major public health challenge to overcome. To accelerate progress towards the goal of ending TB by 2025, 5 years ahead of the global SDG target, it is imperative to outline the incidence and mortality trends of tuberculosis in India. This study aims to provide deep insights into the recent trends of TB incidence and mortality in India from 1990 to 2019.

**Methods:**

This is an observational study based on the most recent data from the Global Burden of Disease (GBD) Study 2019. We extracted numbers, age-specific and age-standardized incidence and mortality rates of Tuberculosis for the period 1990–2019 from the Global Health Data Exchange. The average annual percent change (AAPC) along with 95% Confidence Interval (CI) in incidence and mortality were derived by joinpoint regression analysis; the net age, period, and cohort effects on the incidence and mortality rates were estimated by using Age–Period–Cohort model.

**Results:**

During the study period, age-standardized incidence and mortality rates of TB in India declines from 390.22 to 223.01 and from 121.72 to 36.11 per 100,000 population respectively. The Joinpoint regression analysis showed a significant decreasing pattern in incidence rates in India between 1990 and 2019 for both male and female; but larger decline was observed in case of females (AAPC: − 2.21; 95% CI: − 2.29 to − 2.12; p < 0.001) as compared to males (AAPC: − 1.63; 95% CI: − 1.71 to − 1.54; p < 0.001). Similar pattern was observed for mortality where the declining trend was sharper for females (AAPC: − 4.35; 95% CI: − 5.12 to − 3.57; p < 0.001) as compared to males (AAPC: − 3.88; 95% CI: − 4.63 to − 3.11; p < 0.001). For age-specific rates, incidence and mortality rates of TB decreased for both male and female across all ages during this period. The age effect showed that both incidence and mortality significantly increased with advancing age; period effect showed that both incidence and mortality decreased with advancing time period; cohort effect on TB incidence and mortality also decreased from earlier birth cohorts to more recent birth cohorts.

**Conclusion:**

Mortality and Incidence of TB decreased across all age groups for both male and female over the period 1990–2019. The incidence as well as mortality was higher among males as compared to females. The net age effect showed an unfavourable trend while the net period effect and cohort effect presented a favourable trend. Aging was likely to drive a continued increase in the mortality of TB. Though the incidence and mortality of tuberculosis significantly decreased from 1990 to 2019, the annual rate of reduction is not sufficient enough to achieve the aim of India’s National Strategic plan 2017–2025. Approximately six decades since the launch of the National Tuberculosis Control Programme, TB still remains a major public health problem in India. Government needs to strengthen four strategic pillars “Detect–Treat–Prevent–Build” (DTPB) in order to achieve TB free India as envisaged in the National Tuberculosis Elimination Programme (2020).

**Supplementary Information:**

The online version contains supplementary material available at 10.1186/s12890-021-01740-y.

## Background

Tuberculosis (TB) is a chronic infectious disease and its persistent morbidity and mortality burden remains one of the major public health challenges in India [1]. It is listed as one of the ten most important causes of death from infectious disease in the world [2]. The global TB report published by the World Health Organization (WHO) indicated that in about 10 million people, fell ill with tuberculosis in the year 2019 [3]. According to WHO estimates, around 2.7 million people developed TB in India and over 400,000 people died due to TB in the year 2017 [4]. By WHO estimates, India accounts for 27% of the global estimated 10 million cases and 25% of the estimated 1.6 million deaths. The global burden of disease analysis estimated the number of incident cases to be 3 million people in the year 2016 [5]. A study based on data from the National family Health Survey (NFHS-4) estimates that the self-reported incidence of TB in India is 304/100,000 [6].

United Nation Sustainable Development Goals (SDGs) and WHO’s End-TB strategy aims to end the global TB epidemic with targets to reduce TB deaths by 95% and to cut new cases by 90% by 2035 globally [7]. India’s National Strategic plan 2017–2025 aims to achieve a TB free India, 5 years ahead of the global elimination plan [8]. India had launched a National Tuberculosis Programme (NTP) in sixties following the epidemiological assessment of the situation during 1955–1958 and has already taken several critical steps to showcase itself as a leader for a TB-free world [9]. Despite these impressive commitments, due to less-than-optimal service delivery and various challenges, it could not make much progress in terms of achieving substantially high cure rates and carries the by-far highest burden of TB and MDR-TB.

The actual prevalence of TB in India is likely to be higher than the available prevalence rates. This may be due to the stigma associated with TB and resulting in underreporting. The other factor may be attributed to undiagnosed TB [6]. A major limitation of current estimates of Prevalence and Incidence of TB in India is that India lacks a national TB prevalence survey [10]. Another limitation is incomplete notification from India’s private health sector which uses enormous quantities of anti-TB medications and therefore, disease burden estimates based on TB notifications data may be underestimated [5]. In order to accelerate progress towards the goal of ending TB by 2025, India needs to strengthen the public–private sector response to TB. Thus, India needs to outline the incidence status and trend of TB in India.

The HIV epidemics have a profound and prolonged impact on TB in Asia and the Pacific [11]. HIV has been identified as a risk factor for progression to active TB. Evidence suggests a linear relationship between TB and HIV/AIDS prevalence especially in Africa. Not only the rapid rise in the prevalence of HIV has led to an increasing burden of TB, but HIV also makes the diagnosis of TB more difficult [12]. Although the prevalence of TB in countries with widespread poverty, unemployment and migration has remained stable, the TB incidence has continued to rise [13]. Research highlights the increase in the caseload of tuberculosis due to HIV/AIDS in east Africa and Southern Africa by five or more times Researchers opine differently on the role of HIV epidemic in the TB situation in India, however, Miranda and colleagues report that with an improvement in the access to ART, the TB cases reduce [14]. They demonstrated an 80% reduction in incident TB in HAART (Highly active antiretroviral therapy) treated compared to ART-naïve HIV-infected person in Brazil. It is expected that after the introduction of RNTCP (Revised National TB Control Program), TB incidence, prevalence and mortality rates in India would have reduced by 5% per year if HIV epidemic hadn’t occurred. William et al. suggested that if HIV-infected TB patients receive ART and TB treatment, RNTCP would result in reversing the increase in TB burden due to HIV [15]. With the initiative of a single window delivery of TB and HIV services and other joint collaborative, the TB related fatalities have reduced by 82% with baseline of 2010 among PLHIV [16].

## Materials and methods

In the twenty-first century, the continuously changing scenario and the dynamic nature of health-related challenges across the globe can be understood from the estimates, trends and dynamics of several relevant measures and indices as offered by the GBD study facilitated by the Institute for Health Metrics and Evaluation (IHME). The GBD study is considered to be one of the most comprehensive and reliable worldwide observational epidemiological studies to date. Together with the data and relevant tools, the study provides powerful resources and evidences by quantifying and comparing progress in several health-related dimensions both within and between countries and regions, and thus the GBD study results provide important insights to clinicians, researchers, and policy makers that ultimately promote accountability, assessment and improve lives worldwide.

The comprehensive methods and models supplemented by useful and convenient tools, developed and perfected by the IHME over the past 2 decades provide the most precise estimates of the prevailing picture of the burden of different diseases, injuries and risk factors across the globe. These comparable estimates are considered widely as the key indicators of disease burden quantification and assessment, including the incidence and mortality rate of TB. The present study utilized the data extracted from the recent GBD study 2019 database to systematically summarize and analyse the Incidence and mortality of TB among HIV negative individuals and its changes up to 2019 for India.

### Data source

The GBD 2019 precisely estimated and quantified each epidemiological indices of interest for 204 countries and territories that were grouped into 21 regions and seven super-regions [17]. The estimates are available for incidence, prevalence, mortality, years lived with disability (YLDs, years of life lost (YLLs, and disability-adjusted life-years (DALYs, for 23 age groups; males, females, and both sexes combined. The database utilized a total of 77 different relevant data sources in order to model the cause of death estimates for tuberculosis in India. The causes of death studies by verbal autopsy (VA, Medical certification of cause of deaths of the country and its states, vital statistics, other surveys on cause of death and published scientific articles are among the key sources of data to model the cause of death due to Tuberculosis in India [17].

The conventional Cause of Death Ensemble model (CODEm) and spatiotemporal Gaussian process regression were utilized to quantify Cause-specific death rates and cause fractions. The detailed description of CODEm was reported in numerous related studies in recent years [18–21]. In order to ensure consistency between incidence, prevalence, remission, excess mortality, and cause-specific mortality for most causes, a Bayesian meta-regression modelling tool, DisMod-MR 2.1 was used. The subsequent Uncertainty intervals (UIs) were reported for every metric using the 25th and 975th ordered 1000 draw values of the posterior distribution.

The case definition includes all forms of TB, including pulmonary TB and extrapulmonary TB, which are bacteriologically confirmed or clinically diagnosed. In this study we considered TB with corresponding ICD 10 codes: A10-A19.9, B90-B90.9, K67.3, K93.0, M49.0 and P37.0. Data sources for the incidence rate and Death of TB was extracted from the publicly available online GHDx (Global Health Data Exchange) query tool produced by the IHME (http://ghdx.healthdata.org/gbd-results-tool) [22]. Finally, the Percentage change and annualized rates of change of the estimates for the above-mentioned indices were reported.

### Join point regression analysis

Joinpoint regression analysis was used to determine the magnitude of time trends in incidence and mortality rates of Tuberculosis by calculating the average annual percent change (AAPC) and its 95% confidence interval (CI) [23]. AAPC was calculated from the various annual percent change (APC) values obtained from the regression analysis by taking the geometrically weighted average of APC’s [24]. The average APC (AAPC) was estimated by using the best model considering maximum 5 joinpoint i.e., 6 segments for the full range of our study periods. This analysis was performed using ‘Joinpoint’ software (Joinpoint Regression Program, version 4.8.0.1, NCI) provided by the US National Cancer Institute.

### Age–period–cohort analysis

Previous research shows that the incidence and mortality of tuberculosis have altered over time all around the world, including in India [25]. However, none of these studies took into account the adjustment of age, therefore the impact of shifting demography is unknown. Furthermore, no extensive investigation of the underlying causes of the temporal trends has been conducted. In demography and epidemiology, the Age–Period–Cohort model is a useful technique for identifying secular changes in disease incidence and mortality rates [26]. Age–period–cohort analysis is an extensively used statistical technique when individual or population are followed over time to explore age, period and cohort effect from the observed age specific tuberculosis incidence and mortality rates. We aimed to investigate the long-term trends of Tuberculosis incidence and mortality in India between 1990 and 2019, examining age, period, and cohort-specific effects by sex with the aid of the Age–Period–Cohort framework, using data from the GBD 2019. The intrinsic estimator approach was utilised to conduct the Age–Period–Cohort analyses to solve the problem of model parameter identification (perfect collinearity of the age, period, and cohort variables) [27]. The Age–Period–Cohort Web Tool was used to obtain estimable parameters. The age effect represents the rates of disease in terms of different age groups. The period effect indicates the changes in outcome over time that affect all ages simultaneously. The Cohort effect reflects the changes in outcome across group of individuals born in the same year or years [27–30]. Holford has proposed that if age, period, and cohort trends are orthogonally decomposed into their linear and nonlinear parts, many useful functions can be estimated [31–33].

The model expression of Age–period–cohort is generally written as

$${\text{Y }} = {\text{ log}}\left( {\text{M}} \right) \, = \, \mu \, + \, \alpha {\text{age}}_{{1}} + \, \beta {\text{period}}_{{1}} + \, \gamma {\text{cohort}}_{{1}} + \, \varepsilon$$

where M stands for the incidence of the corresponding age group, µ stands for the intercept item, α, β, and γ stand for the corresponding age, period and cohort effect, and "ε" is the random error. It has age_1_ = period_1_ − cohort_1_ [34]

To estimate the parameters and functions Age–period–cohort Web Tool use the statistical method of weighted least square and the assumption that the count data follow a Poisson distribution allowing for extra-Poisson variation.

In the present study, we focussed on a number of estimable functions that we have discussed in this section [35]. The longitudinal Age Curve indicates the fitted longitudinal age-specific rates in reference to cohort adjusted for period deviations [36]. The period (or cohort) RR indicates the ratio of the age-specific rate in each period (or cohort) relative to the reference period (or cohort) [35]. Further local drift and net drift are two other important parameters in the Age–period–cohort model. The local drift (or local net) indicates the (overall) log linear trend by calendar period and birth cohort for each age group and is analogous to the (overall) annual percentage change [37]. Wald Chi-Square tests were adopted to test the significance of the estimable parameters and functions, and p-values less than 0.05 were considered for statistical significance [38].

We obtained the estimable parameters by the Age–period–cohort Web Tool [35] (Biostatistics Branch, National Cancer Institute, Bethesda, MD, USA). Correspondence to the requirement of the Age–Period–Cohort Web Tool the incidence (or mortality) and population data of tuberculosis were arranged into consecutive 5-year periods from 1990 to 2019 (1990–94, 1995–99, …, 2010–14, 2015–19) and successively incidence and mortality data into 18 five year age groups (5–9, 10–14, …, 85–89, 90–94), spanning 23 partially overlapping 5-year birth cohorts (1900–04, 1905–09, …, 2010–14). Furthermore, the central period, and central birth cohort were defined as the references in all analyses. The central period and birth cohort has been chosen depending on two major reasons. Firstly, the National TB Programme (NTP) was launched in the early sixties (1962) by the Government of India. Secondly, DOTS was officially launched as the RNTCP strategy in 1997, and by the end of 2005 the entire country was covered under the programme. These are the reasons for choosing mid cohort (1955–59) as a reference cohort and mid period (2000–04) as a reference period for seeing the before and after effect of these programmes.

## Results

### Descriptive analysis of incidence and mortality trends of tuberculosis

**Fig. 1:**
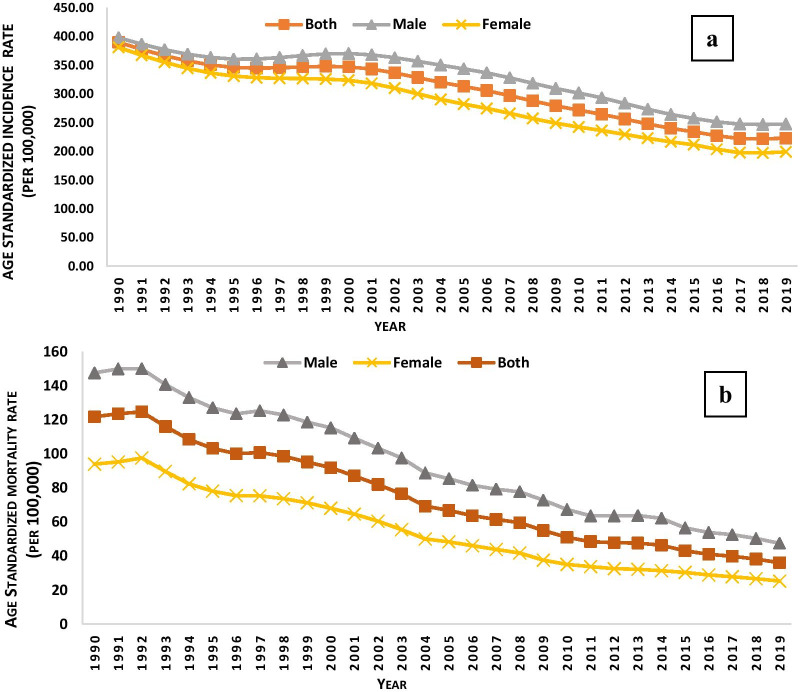
Trends in the age-standardized rates for Tuberculosis in male, female and both sexes from 1990 to 2019; **a** incidence, **b** mortality

Trends in the age-standardized incidence rate (ASIR) and age-standardized mortality rate (ASMR) in male, female and both sexes at all ages for tuberculosis from 1990 to 2019 are depicted in Fig. 1a, b respectively. For all years both age-standardized Incidence and mortality rate of TB was higher among males as compared to females. The age-standardized incidence rates for both sexes of tuberculosis decreased from 1990 to 1996, increased in the period 1997—1999 and then again continuously decreased till 2018. Age-standardized mortality rates experienced a slight increase and decrease over the years for male and female. Overall, the mortality rate due to tuberculosis largely decreased in 2019 (36.11 deaths per 100,000) as in for 1990 (121.72 deaths per 100,000).

#### Trends in age standardized incidence and mortality rate for male, female and both sexes

**Fig. 2:**
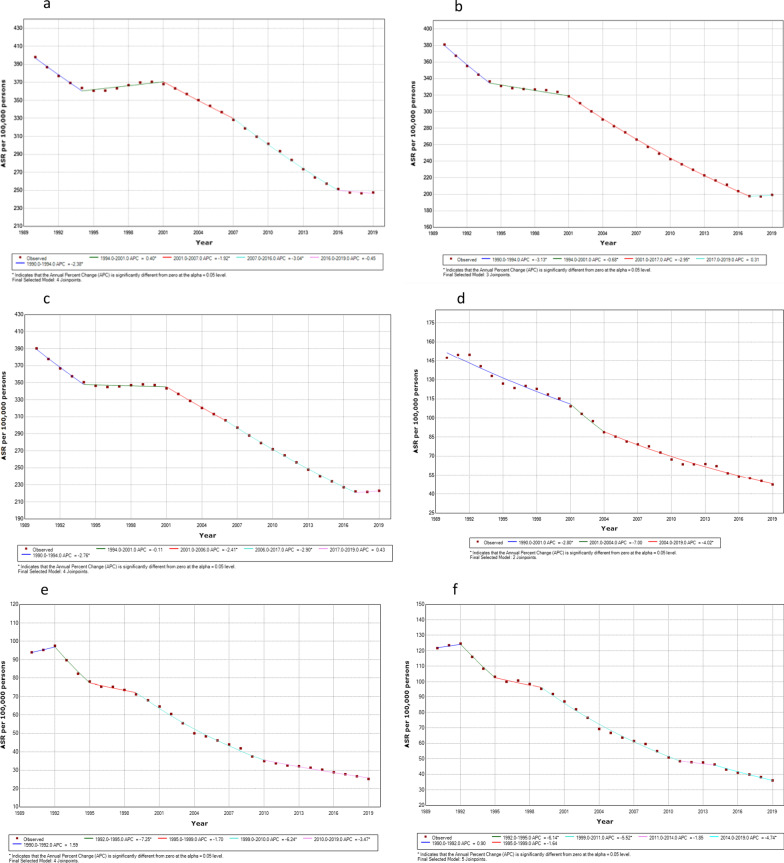
Sex-specific temporal trends in age standardised incidence and mortality of tuberculosis in India based on the joinpoint regression analysis (1990–2019). *Note*: **a** Incidence male, **b** incidence female, **c** incidence both sexes, **d** mortality male, **e** mortality female, **f** mortality both sexes

Figure 2 and Additional file 1: Table 1 shows APC and AAPC of TB incidence and mortality in India from 1990 to 2019 for male, females and both sexes combined. The age standardized TB incidence rate in India between 1990 and 2019 ranged from 390.22 to 223.01 per 100,000 population. The regression model showed a significant decreasing pattern in incidence rate in India between 1990 and 2019 for both male and female; but larger decline is observed in case of females (AAPC: − 2.21; 95% CI: − 2.29 to − 2.12; p < 0.001) as compared to males (AAPC: − 1.63; 95% CI: − 1.71 to − 1.54; p < 0.001). The overall incidence rate for both sexes combined has decreased significantly over the period (AAPC: − 1.90; 95% CI: − 1.97 to − 1.83; p < 0.001). Similarly, the mortality rate due to tuberculosis has largely declined in 2019 (36.11 deaths per 100,000) as compared to 1990 (121.72 deaths per 100,000). The regression analysis also showed decreasing trend in the age standardized mortality rates for period 1990 to 2019 (AAPC: − 4.11; 95% CI: − 5.03 to − 3.18; p < 0.001); however the declining trend was sharper for female (AAPC: − 4.35; 95% CI: − 5.12 to − 3.57; p < 0.001) as compared to male (AAPC: − 3.88; 95% CI: − 4.63 to − 3.11; p < 0.001).

**Table 1:** Average Annual Percent Change (AAPC) of tuberculosis incidence and mortality by age and gender from 1990 to 2019 using Joinpoint Regression Analysis

Age-group (year)	Incidence (95% CI)		Mortality (95% CI)	
	Male	Female	Male	Female
5–9	− 4.16 (− 4.28, − 4.04)	− 4.77 (− 4.99, − 4.55)	− 6.85 (− 8.67, − 5.01)	− 8.34 (− 9.52, − 7.13)
10–14	− 2.56 (− 2.87, − 2.25)	− 3.53 (− 3.65, − 3.40)	− 5.22 (− 6.13, − 4.31)	− 6.11 (− 8.91, − 3.22)
15–19	− 1.37 (− 1.59, − 1.15)	− 2.54 (− 2.72, − 2.37)	− 4.68 (− 7.75, − 1.51)	− 5.61 (− 7.13, − 4.06)
20–24	− 1.57 (− 1.69, − 1.45)	− 2.78 (− 2.91, − 2.65)	− 3.98 (− 5.47, − 2.46)	− 5.45 (− 7.01, − 3.86)
25–29	− 1.13 (− 1.30, − 0.95)	− 2.50 (− 2.68, − 2.31)	− 3.31 (− 4.09, − 2.53)	− 4.82 (− 6.23, − 3.39)
30–34	− 1.02 (− 1.17, − 0.86)	− 2.10 (− 2.26, − 1.93)	− 3.30 (− 4.39, − 2.20)	− 4.47 (− 5.56, − 3.37)
35–39	− 1.03 (− 1.19, − 0.87)	− 1.63 (− 1.74, − 1.51)	− 3.07 (− 3.85, − 2.28)	− 4.08 (− 4.78, − 3.37)
40–44	− 1.13 (− 1.23, − 1.03)	− 1.38 (− 1.46, − 1.29)	− 3.44 (− 3.74, − 3.13)	− 3.92 (− 5.16, − 2.66)
45–49	− 1.41 (− 1.55, − 1.27)	− 1.29 (− 1.75, − 0.82)	− 3.58 (− 4.59, − 2.56)	− 4.13 (− 4.85, − 3.41)
50–54	− 1.55 (− 1.73, − 1.38)	− 1.44 (− 1.81, − 1.07)	− 3.78 (− 4.74, − 2.81)	− 3.61 (− 4.56, − 2.66)
55–59	− 1.53 (− 1.63, − 1.43)	− 1.70 (− 1.89, − 1.51)	− 3.45 (− 4.51, − 2.38)	− 4.03 (− 5.06, − 2.99)
60–64	− 1.80 (− 1.89, − 1.72)	− 1.90 (− 2.07, − 1.73)	− 4.09 (− 4.85, − 3.33)	− 4.31 (− 4.95, − 3.65)
65–69	− 2.37 (− 2.49, − 2.25)	− 2.41 (− 2.63, − 2.19)	− 4.10 (− 5.26, − 2.93)	− 4.40 (− 5.82, − 2.96)
70–74	− 2.49 (− 2.57, − 2.41)	− 2.56 (− 2.85, − 2.27)	− 3.96 (− 4.85, − 3.05)	− 4.38 (− 5.53, − 3.21)
75–79	− 2.35 (− 2.55, − 2.16)	− 2.41 (− 2.74, − 2.09)	− 3.93 (− 4.79, − 3.05)	− 4.53 (− 5.91, − 3.13)
80–84	− 2.29 (− 2.47, − 2.11)	− 2.40 (− 2.73, − 2.08)	− 3.74 (− 4.76, − 2.71)	− 4.26 (− 5.73, − 2.77)
85–89	− 2.34 (− 2.66, − 2.01)	− 2.36 (− 2.63, − 2.10)	− 4.20 (− 5.38, − 3.00)	− 4.74 (− 6.54, − 2.89)
90–94	− 2.34 (− 2.63, − 2.04)	− 2.34 (− 2.62, − 2.07)	− 4.98 (− 6.32, − 3.62)	− 4.65 (− 5.65, − 3.63)

AAPC, average annual percent change; CI, confidence interval; ASR, age standardised rates

#### Trends in age-specific incidence and mortality rates using joinpoint regression analysis

Table 1 depicts the average annual percent change (AAPC) in tuberculosis incidence and mortality for both male as well as female in India from 1990 to 2019. For age-specific rates, incidence and mortality rates of TB decreased for both male and female across all ages during the period. Sharper decline was observed in the initial age groups and older age groups for both male and female in the age specific incidence and mortality.

### The age, period, cohort effects of incidence and mortality rate from tuberculosis

**Fig. 3:**
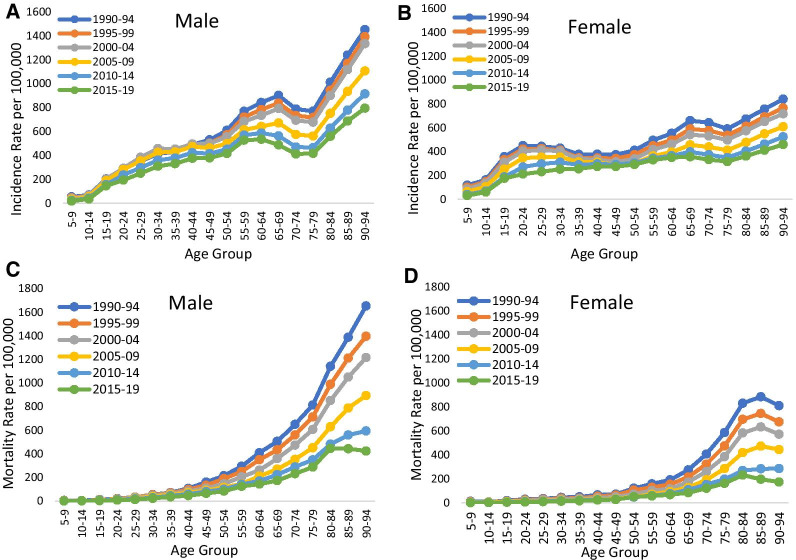
Age-specific tuberculosis incidence and mortality for India in 1990–2019

The existence of a period effect on the incidence rate of tuberculosis (A, B) and mortality rate (C, D) for male and female from 1990 to 2019 are depicted in Fig. 3. In case of tuberculosis incidence, period effect did not show much variation up to age 14 year for both male and female. Incidence rate (Fig. 3a, b) of TB was higher among male than female in all ages. Lower incidence rate of TB was observed in later period (2015–19) than earlier period (1990–94) for both male and female. Overall, the incidence of TB increased with age group from 10–14 to 65–69 and slightly decreased from ages 65–69 to 75–79 and again increased with high pace after the age group 75–79 for male. However, female incidence also increased but with a slower pace after the age 75–79 year. In case of mortality rate of TB (Fig. 3c, d) period effect did not reflect clear variability up to age 25–29 year for male and 45–49 year for female. Mortality rate were higher among male than female in all ages. It was also evident that within the same period older males and females had higher mortality. In a particular period, mortality rate of TB increased with high pace for male in all ages while with a slower pace among female.

**Fig. 4:**
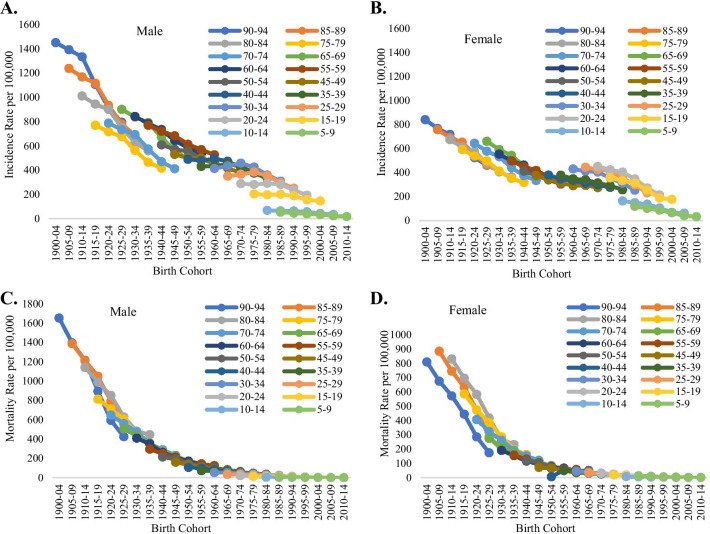
Cohort-based variation in age-specific Tuberculosis incidence and mortality for India in 1990–2019

Figure 4a–d suggested the existence of a cohort effect of incidence and mortality of tuberculosis in India 1990–2019. Earlier birth cohort (1990–94) had higher incidence and mortality rate than later birth cohort (2010–14) and within the same age group incidence and mortality decreased from older cohort to newer cohort for male and female both. Within a particular age, newer birth cohort had lower incidence and mortality among both male and female. For instance, among males in the age group 90–94 years incidence was found to be higher for the birth cohort (1900–04) with reference to the birth cohort (1925–29). It was also clearly depicted from the figure that males had approximately double incidence and mortality of tuberculosis in the older birth cohorts.

**Fig. 5:**
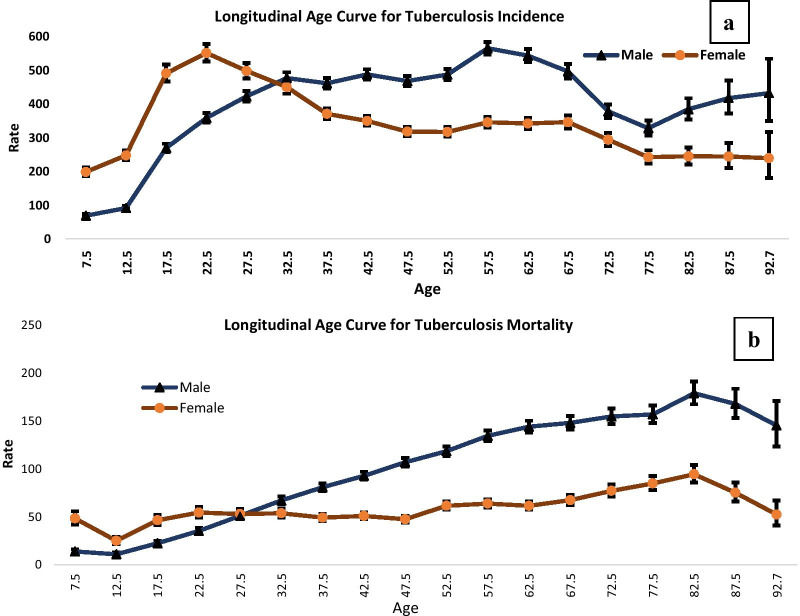
Longitudinal age curve of tuberculosis incidence and mortality rate under the Age–Period–Cohort framework; **a** incidence, **b** mortality

The longitudinal age curves of tuberculosis incidence and mortality by sex are depicted in Fig. 5a, b respectively. In the same birth cohort, incidence rate of tuberculosis substantially increased in the ages 5–9 to 20–24 and after that it decreased in all remaining age groups for female.

In the context of incidence, a very uneven pattern was observed among males. In early ages (before 30–34 years) tuberculosis incidence was higher among females within the same birth cohort. Thus, females were at a better position in the context of Tuberculosis incidence. In the same birth cohort mortality rate of tuberculosis increased for all ages except in the age 5–9 years and started declining from age 80–84 years for both sexes. Within the same birth cohort males had higher risk of tuberculosis mortality which was approximately similar in all birth cohorts except the earlier cohort for instance before age 25-29 years. In both the sexes tuberculosis mortality was highest for the age 80- 84 years.

The period effect RR of tuberculosis incidence and mortality for both sexes are depicted in Table 2. Through the years 1990–2019 of observation, the RR of tuberculosis Incidence and mortality decreased for both sexes. With respect to the reference category (2000–04) mortality due to tuberculosis among female decreased by 53 percent which was higher than male (49 percent) in (2015–2019).

**Table 2:** Period RRs of tuberculosis incidence and mortality rate adjusted for age and birth cohort effects compared to the reference period (2000–04) and the corresponding 95% CI

Period	Incidence		Mortality	
	Male	Female	Male	Female
1990–94	1.04 (1.02–1.07)	1.16 (1.13–1.19)	1.37 (1.33–1.41)	1.56 (1.49–1.63)
1995–99	1.00 (0.98–1.03)	1.06 (1.03–1.09)	1.20 (1.16–1.24)	1.26 (1.21–1.32)
2000–04	1.0 (1.0–1.0)	1.0 (1.0–1.0)	1.0 (1.0–1.0)	1.0 (1.0–1.0)
2005–09	0.90 (0.88–0.92)	0.86 (0.84–0.88)	0.78 (0.75–0.80)	0.72 (0.69–0.75)
2010–14	0.77 (0.75–0.78)	0.75 (0.73–0.77)	0.63 (0.61–0.66)	0.56 (0.53–0.59)
2015–19	0.67 (0.65–0.69)	0.68 (0.66–0.70)	0.51 (0.49–0.53)	0.47 (0.45–0.5)

The cohort effect RR of tuberculosis incidence and mortality for both sexes are depicted in Table 3. Among all 23 cohort’s mid cohort 1955–59 was taken as a reference cohort. The incidence and mortality risk continuously decreased from 1900–04 to 2010–14 birth cohort which show the presence of cohort effect. Highest percentage decrement in incidence and mortality was observed among females.

**Table 3:** Cohort RRs of tuberculosis incidence and mortality rate adjusted for age and period effects compared to the referent cohort (1955–59) and the corresponding 95% CI

Cohort	Incidence		Mortality	
	Male	Female	Male	Female
1900–04	3.57 (1.71–7.42)	3.59 (1.23–10.5)	11.8 (7.60–18.6)	15.6 (7.64–32.1)
1905–09	3.17 (2.38–4.23)	3.18 (2.13–4.74)	8.78 (7.31–10.5)	12.0 (9.28–15.6)
1910–14	2.81 (2.38–3.31)	2.82 (2.28–3.50)	6.85 (6.13–7.64)	9.18 (7.91–10.6)
1915–19	2.48 (2.21–2.79)	2.51 (2.16–2.91)	5.50 (5.06–5.98)	7.24 (6.45–8.12)
1920–24	2.19 (2.01–2.39)	2.23 (2.01–2.48)	4.44 (4.15–4.75)	5.60 (5.07–6.18)
1925–29	1.92 (1.80–2.05)	1.95 (1.80–2.12)	3.55 (3.35–3.77)	4.21 (3.85–4.61)
1930–34	1.67 (1.59–1.76)	1.69 (1.58–1.81)	2.88 (2.73–3.03)	3.18 (2.93–3.45)
1935–39	1.46 (1.39–1.52)	1.47 (1.39–1.56)	2.33 (2.21–2.44)	2.47 (2.29–2.67)
1940–44	1.28 (1.23–1.34)	1.30 (1.23–1.38)	1.83 (1.74–1.91)	1.95 (1.81–2.10)
1945–49	1.15 (1.11–1.2)	1.17 (1.11–1.23)	1.51 (1.44–1.58)	1.59 (1.48–1.71)
1950–54	1.06 (1.02–1.09)	1.06 (1.01–1.11)	1.17 (1.12–1.22)	1.23 (1.15–1.32)
1955–59	1.0 (1.0–1.0)	1.0 (1.0–1.0)	1.0 (1.0–1.0)	1.0 (1.0–1.0)
1960–64	0.94 (0.91–0.97)	0.94 (0.90–0.98)	0.86 (0.82–0.90)	0.81 (0.75–0.87)
1965–69	0.90 (0.87–0.93)	0.90 (0.86–0.94)	0.74 (0.70–0.78)	0.69 (0.64–0.74)
1970–74	0.87 (0.84–0.90)	0.85 (0.81–0.89)	0.64 (0.61–0.68)	0.54 (0.50–0.59)
1975–79	0.82 (0.79–0.85)	0.77 (0.74–0.81)	0.54 (0.51–0.58)	0.43 (0.39–0.47)
1980–84	0.76 (0.73–0.79)	0.69 (0.66–0.72)	0.43 (0.40–0.46)	0.32 (0.29–0.35)
1985–89	0.71 (0.68–0.74)	0.60 (0.57–0.63)	0.36 (0.33–0.39)	0.25 (0.22–0.28)
1990–94	0.65 (0.62–0.68)	0.49 (0.47–0.52)	0.28 (0.25–0.31)	0.18 (0.16–0.21)
1995–99	0.57 (0.54–0.60)	0.39 (0.37–0.42)	0.21 (0.18–0.24)	0.12 (0.10–0.14)
2000–04	0.53 (0.50–0.57)	0.33 (0.31–0.35)	0.15 (0.12–0.19)	0.08 (0.07–0.10)
2005–09	0.38 (0.34–0.43)	0.22 (0.20–0.25)	0.10 (0.07–0.14)	0.05 (0.04–0.07)
2010–14	0.27 (0.22–0.33)	0.15 (0.13–0.18)	0.06 (0.03–0.10)	0.02 (0.01–0.04)

**Fig. 6:**
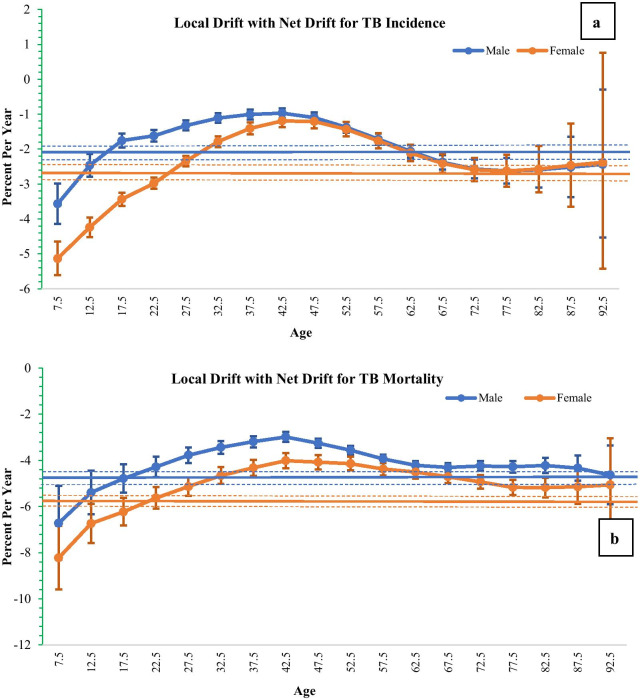
Local drift with net drift with 95% confidence intervals in tuberculosis incidence and mortality by gender; **a** incidence, **b** mortality

Figure 6a, b show the net drifts and local drifts for Tuberculosis incidence and mortality in India among both sexes from 1990 to 2019 respectively. The net drift values are an overall estimated annual percentage change, and local drift values are an estimated annual percentage change values for each age group. From 1990 through 2019, we found that the net drifts for tuberculosis incidence per year were − 1.77% (95% Confidence Interval, − 1.87% to − 1.66%) for male and − 2.16% (95% Confidence Interval, − 2.30% to − 2.03%) for female. The net drifts for tuberculosis mortality per year were − 3.96% (95% Confidence Interval, − 4.09% to − 3.83%) for male and − 4.83% (95% Confidence Interval, − 5.00% to − 4.67%) for female. The local drift value was found to be below 0 in all age groups in both sexes which was lowest in age 5–9 and highest in age 40–44. The local drift values increased with higher age groups and started declining after 40–44 age. In addition, the results of Wald test demonstrated that for incidence and mortality, cohort and period RRs and the net drifts and local drifts were statistically significant at p < 0.05 (Additional file 1: Table 2).

## Discussion

This study utilizes the GBD database to analyze the incidence and prevalence of Tuberculosis in the period 1990–2019 in India. The analysis showed that there has been a decline in the age standardized incidence rate in the period 1990–2019 among both sexes. However, mortality rates experienced a zig-zag pattern when standardized for age among males and females. The overall mortality rate declined from 122 deaths per 100,000 to 36 per 100,000 in the period 1990–2019. The long-term trends in incidence of tuberculosis were analyzed using Joinpoint regression. The regression analysis also showed a decreasing trend in the age standardized mortality rates for the period 1990–2014 and the AAPC was found to be − 4.1. The results obtained from the Age–Period–Cohort analysis showed that the incidence and mortality due to tuberculosis in India decreased with the recent cohort. Studies have found that the trend of tuberculosis is related to age and period [39].

The results from the Age–Period–Cohort model as obtained in India have a similar trend as that of China. A study which utilized the Age–Period–Cohort -model to distinguish age, period and cohort effect in China, India and the United States found that period effect showed a decline in the incidence of tuberculosis in all of the three countries [40]. Further, it found an increase in the incidence of tuberculosis in most of the age-group. The application of Age–Period–Cohort modelling in the study of tuberculosis incidence has been described by Wu and Colleagues [41].

**Age effect:** The rates of disease at different ages are presented by the age effect. It is known that the human age is associated with the disease. Study findings shows that in India incidence and mortality were higher in the older age groups. It is also found that in most of the ages (except some younger ages in case of incidence) females were having lower value of incidence and mortality per 100,000 population. The higher prevalence of tuberculosis among older population is attributed to poor nutrition and socio-economic status [42, 43]. Age standardized incidence and mortality rates of tuberculosis was found to be higher among males as compared to females at all ages. With the increase in the age, the incidence rate increased. This is attributed to the population ageing higher in older age group. With the increase in the age, people are at higher risk of developing disease such as diabetes [44] and indoor air pollution [45]. Vulnerable population are more prone to developing tuberculosis. A study by Varun has estimated that the reported annual TB incidence in India will decrease slightly [45]. Cui et al. have reported that the age effect of the 35–60 years age group continued to increase and decreased slightly in the 60–70 age group [40]. Further, it was found that the rapid rise of age RRs in India is attributable to alcohol consumption in the age group 35–60. The risk factor for tuberculosis is alcohol consumption, and its related problems [46]. Alcohol consumption is stated to be a significant risk factor of tuberculosis in various countries. With the upcoming ageing in the country, India will be facing challenges in the prevention and control of tuberculosis. The weak immune system, malnutrition and other physiological changes will increase the risk of tuberculosis in the older population and thus the TB burden among the senior citizens will be higher than the other age groups.

**Period effect:** Period effect was not found of much significance up to the age of 14 years for both sexes in case of incidence of tuberculosis. From the available information, it was evident that the period RRs of incidence had a downward trend in India in the period 1990–2019. The factors associated with the decline in the RRs might be due to improved living conditions, increased accessibility and availability of good health facilities and increasing awareness towards sanitation and hygiene practices. Coupled with the improvement in India’s public health facility and strategic programs aimed at eliminating tuberculosis is the reason behind declining RRs in India. Earlier period (1990–94) had higher incidence rates of tuberculosis than in the later period 2015–19. This was the same for both male and females. Cui et. al. in his study reports that the period RRs in India has declined from 1992 to 2017 [40]. At some period, the incidence of TB showed an increasing pattern with a substantially faster pace after the 75-79 years for males. However, for females the increase in incidence was not much pronounced after the age 75–79. As far as mortality is concerned, period effect was not much visible up to the age 25–29 for males and 45–49 for females. Mortality rates were higher for males than females for all ages. The introduction of the 1993 policy to control the TB in India has been proved successful in the improved treatment of TB which is estimated to avoid 200,000 deaths [47]. The TB control program has been quite effective in reducing the incidence of tuberculosis and further preventing its speed.

**Cohort effect:** Study highlights the existence of a cohort effect in the incidence and mortality due to tuberculosis in India in the period 1990–2019. Differences in the cohort RRs in the birth cohort usually apply to the balance between new infections and compromised immune responses to previous infections [48]. The cohort effect on the incidence of tuberculosis was found to be higher among 1990–94 birth cohorts than in the later birth cohorts 2010–14. Similarly, the cohort effect on the mortality due to tuberculosis in the period 1994–2014 showed a declining trend in India. In the same age group, the tuberculosis incidence and mortality decreased from older cohorts to newer birth cohorts for both males and females. Analysis from a long-term trend study in incidence of tuberculosis has also found a declining trend in the incidence from 1908 to 1962 and from 1987–1997 [40]. TB in India is mainly considered to have originated from poverty. Unavailability and inaccessibility to food will lead to malnutrition in the majority of Indians [49]. Increasing population and rapid urbanization would result in TB risk factors in the country [50]. The most prevalent risk factors for tuberculosis in India are AIDS, smoking and drinking and diabetes [51]. Study outlines that male have approximately double the incidence and mortality of tuberculosis in the older birth cohorts. The decline in the cohort RRs signifies the effective measures taken to reduce the burden of tuberculosis in the country.

The longitudinal age curve of tuberculosis showed that incidence rate of tuberculosis among females had a substantial increase in the age 5 to 24 years and then decreased thereafter in all ages in the same birth cohorts. Mortality due to tuberculosis was found to be highest among population aged 80–84 years. While considering the period effect, the relative risk of tuberculosis incidence and mortality decreased for both sexes in the period 1990–2019. Considering period 2000- 04 as referent, mortality due to tuberculosis decreased by 53 percent and 49 percent among females and males respectively in the period 2015–19. Cohort RRs showed that the incidence and mortality of tuberculosis decreased from 1900–2004 to 2010–14 for males and females. This decrement was higher for females. Overall, the annual net drift for tuberculosis incidence was − 1.77 for male and − 2.16 for females. Similarly, for mortality due to tuberculosis the net drift values were − 3.96 for males and − 4.83 for females. The local drift values initially increased with higher age groups and then followed a decreasing pattern after 40–44 age group.

Recognizing that TB largely depends on the social and economic determinates of health, the change in overall trend of the tuberculosis in India is mainly driven by trends in demography, epidemiology or socio-economic factors. Areas of socio-economic deprivation are present throughout the country. India TB report estimates that the majority of TB burden is among the working age-group [16]. Uttar Pradesh is the largest contributor of TB cases in the country; however, the highest notification comes from Delhi and Chandigarh [16]. Looking at the epidemiological transition in the country, the disease burden and the risk factors trends for each state of the country vary. Likewise, the social development status of the status in India also varies. Moreover, wider determinants of health, housing quality and availability, differences in health seeking behaviour and access to good quality education also have a great effect on the determining the health status of an individual. The noticeable change in the government health policies, introduction of RNTCP, improved sanitation and hygiene level and administrative and political commitments are responsible for reducing the mortality of tuberculosis in the country. Significant gains were made over the last National Strategic Plan (NSP 2012–17). Financial incentives, strengthened health systems, contact investigating, address to poverty, malnutrition aimed at eliminating TB in India are the possible reasons for the observed period and cohort effects.

Our study has several limitations. First, Although the GBD study incorporates methods to adjust for incomplete or missing data and quality of the data, there may still be the possibility of some inaccuracy in the mortality data. Second, we have performed Age–Period–Cohort analysis in periods of multiples of five years as GBD provides data in five-year intervals that may lead to the smoothening of certain subtle variations in age, period and cohort effects. Third, being an ecological study, the interpretations derived here are true at population levels but do not necessarily hold for individuals.

## Conclusions

This study shows that overall incidence and mortality of tuberculosis in all ages has decreased in the last three decades in India. The incidence as well as mortality was higher among males as compared to females during study period. In India age standardized incidence and mortality rates of tuberculosis significantly decreased from 1990 to 2019. The incidence and mortality rates of tuberculosis increased with advancing age whereas it decreased with advancing period and earlier to later birth cohort for both male and female.

Though the incidence and mortality of tuberculosis significantly decreased from 1990 to 2019, but the annual rate of reduction is not sufficient enough to achieve the aim of India’s National Strategic plan 2017–2025. Approximately six decades since the launch of the National Tuberculosis Control Programme, TB still remains a major public health problem in India. Government needs to strengthen four strategic pillars “Detect–Treat–Prevent–Build” (DTPB) to achieve TB free India.

## Supplementary Information

**Additional File 1: Table 1.** Sex-specific temporal trends in incidence and mortality of tuberculosis in India based on the joinpoint regression analysis (1990-2019).** Table 2.** Wald χ2 tests to test the significance of estimable functions.

## Data Availability

The datasets analyzed during the current study are available in the (Global Health Data Exchange) query tool produced by the IHME repository, [http://ghdx.healthdata.org/gbd-results-tool].

## Abbreviations

AAPC: Average Annual Percent change
APC: Annual Percent change
ASIR: Age Standardized Incidence Rate
ASMR: Age Standardized Mortality Rate
CODEm: Cause of Death Ensemble modelling tool
DALY: Disability-adjusted life year
GBD: Global Burden of Disease (study)
GHDx: Global Health Data exchange
ICD: International Classification of Disease
MDR-TB: Multi Drug Resistant Tuberculosis
NFHS: National Family Health Survey
NTP: National Tuberculosis Programme
RR: Relative Risk
SDG: Sustainable Development Goals
TB: Tuberculosis
UI: Uncertainty interval
WHO: World Health Organization
YLDs: Years lived with disability
YLLs: Years of life lost

## References

[1] Pai M, Correa N, Mistry N, Jha P (2017). Reducing global tuberculosis deaths—time for India to step up. Lancet.

[2] World Health Organization (2013). Global tuberculosis report 2013.

[3] World Health Organization. Global TB Report. 2019. https://www.who.int/tb/publications/global_report/en/. Accessed 1 Mar 2021.

[4] World Health Organization (2017). Global tuberculosis report 2018.

[5] Arinaminpathy N, Batra D, Khaparde S, Vualnam T, Maheshwari N, Sharma L, Dewan P (2016). The number of privately treated tuberculosis cases in India: an estimation from drug sales data. Lancet Infect Dis.

[6] Mazumdar S, Satyanarayana S, Pai M (2019). Self-reported tuberculosis in India: evidence from NFHS-4. BMJ Glob Health.

[7] Uplekar M, Weil D, Lonnroth K, Jaramillo E, Lienhardt C, Dias HM, Gilpin C (2015). WHO's new end TB strategy. Lancet.

[8] Central TB Division, Directorate General of Health services, Ministry of Health and Family Welfare, Nirman Bhavan ND. National strategic plan for tuberculosis elimination 2017–2025. 2017.

[9] Indian Council of Medical Research. Tuberculosis Sub-Committee. Tuberculosis in India: a sample survey, 1955–58. 1959.

[10] Onozaki I, Law I, Sismanidis C, Zignol M, Glaziou P, Floyd K (2015). National tuberculosis prevalence surveys in Asia, 1990–2012: an overview of results and lessons learned. Trop Med Int Health.

[11] Narain JP, Lo YR (2004). Epidemiology of HIV-TB in Asia. Indian J Med Res.

[12] Narain JP, Raviglione MC, Kochi A (1992). HIV-associated tuberculosis in developing countries: epidemiology and strategies for prevention. Tuber Lung Dis.

[13] Swaminathan S, Narendran G (2008). HIV and tuberculosis in India. J Biosci.

[14] Miranda A, Morgan M, Jamal L, Laserson K, Barreira D, Silva G, Santos J, Wells C, Paine P, Garrett D (2007). Impact of antiretroviral therapy on the incidence of tuber-culosis: the Brazilian experience, 1995–2001. PLoS ONE.

[15] Williams BG, Granich R, Chauhan LS, Dharmshaktu NS, Dye C (2005). The impact of HIV/AIDS on the control of tuberculosis in India. Proc Natl Acad Sci.

[16] India TB report. Revised National TB control programme, Central TB Division, MOHFW, India. 2019.

[17] Vos T, Lim SS, Abbafati C, Abbas KM, Abbasi M, Abbasifard M, Abbasi-Kangevari M, Abbastabar H, Abd-Allah F, Abdelalim A, Abdollahi M, Bhutta ZA (2020). Global burden of 369 diseases and injuries in 204 countries and territories, 1990–2019: a systematic analysis for the Global Burden of Disease Study 2019. Lancet.

[18] Lozano R, Naghavi M, Foreman K, Lim S, Shibuya K, Aboyans V, Abraham J, Adair T, Aggarwal R, Ahn SY, AlMazroa MA, Remuzzi G (2012). Global and regional mortality from 235 causes of death for 20 age groups in 1990 and 2010: a systematic analysis for the Global Burden of Disease Study 2010. Lancet.

[19] Foreman KJ, Lozano R, Lopez AD, Murray CJ (2012). Modeling causes of death: an integrated approach using CODEm. Popul Health Metr.

[20] Murray CJ, Ezzati M, Flaxman AD, Lim S, Lozano R, Michaud C, Naghavi M, Salomon JA, Shibuya K, Vos T, Wikler D, Lopez AD (2012). GBD 2010: design, definitions, and metrics. Lancet.

[21] Murray CJ, Ortblad KF, Guinovart C, Lim SS, Wolock TM, Roberts DA, Dansereau EA, Graetz N, Barber RM, Brown JC, Wang H, Jacobsen KH (2014). Global, regional, and national incidence and mortality for HIV, tuberculosis, and malaria during 1990–2013: a systematic analysis for the Global Burden of Disease Study 2013. Lancet.

[22] Global Burden of Disease Collaborative Network. Global Burden of Disease Study 2019 (GBD 2019) Results. Seattle: Institute for Health Metrics and Evaluation (IHME); 2020. Available from http://ghdx.healthdata.org/gbd-results-tool

[23] Kim HJ, Fay MP, Feuer EJ, Midthune DN (2000). Permutation tests for joinpoint regression with applications to cancer rates. Stat Med.

[24] Clegg LX, Hankey BF, Tiwari R, Feuer EJ, Edwards BK (2009). Estimating average annual per cent change in trend analysis. Stat. Med..

[25] Dye C, Lönnroth K, Jaramillo E, Williams BG, Raviglione M (2009). Trends in tuberculosis incidence and their determinants in 134 countries. Bull World Health Organ.

[26] Yang Y, Fu WJ, Land KC (2004). A methodological comparison of age–period–cohort models: the intrinsic estimator and conventional generalized linear models. Sociol Methodol.

[27] Yang Y, Land KC (2013). Age–period–cohort analysis: New models, methods, and empirical applications.

[28] Robertson C, Gandini S, Boyle P (1999). Age–period–cohort models: a comparative study of available methodologies. J Clin Epidemiol.

[29] Wang Z, Hu S, Sang S, Luo L, Yu C (2017). Age–period–cohort analysis of stroke mortality in China: data from the Global Burden of Disease Study 2013. Stroke.

[30] Xiaxue 2020: Age–period–cohort analysis of kidney cancer deaths attributable to high body-mass index in China and U.S. adults. 2020. 10.1186/s12889-020-09007-7 PMC7281955 32513130

[31] Holford TR (1983). The estimation of age, period and cohort effects for vital rates. Biometrics.

[32] Yang HP, Anderson WF, Rosenberg PS, Trabert B, Gierach GL, Wentzensen N, Cronin KA, Sherman ME (2013). Ovarian cancer incidence trends in relation to changing patterns of menopausal hormone therapy use in the United States. J Clin Oncol.

[33] Pastor-Barriuso R, López-Abente G (2014). Changes in period and cohort effects on haematological cancer mortality in Spain, 1952–2006. BMC Cancer.

[34] Luo L (2013). Assessing validity and application scope of the intrinsic estimator approach to the age–period–cohort problem. Demography.

[35] Rosenberg PS, Check DP, Anderson WF (2014). A web tool for age–period–cohort analysis of cancer incidence and mortality rates. Cancer Epidemiol Prev Biomarkers.

[36] Anderson WF, Rosenberg PS, Menashe I, Mitani A, Pfeiffer RM (2008). Age-related crossover in breast cancer incidence rates between black and white ethnic groups. JNCI J Natl Cancer Inst.

[37] Chaturvedi AK, Anderson WF, Lortet-Tieulent J, Curado MP, Ferlay J, Franceschi S, Rosenberg PS, Bray F, Gillison ML (2013). Worldwide trends in incidence rates for oral cavity and oropharyngeal cancers. J Clin Oncol.

[38] Ding Y, Zhou J, Yang J, Laflamme L (2017). Demographic and regional characteristics of road traffic injury deaths in Jiangsu Province, China. J Public Health.

[39] Vynnycky E, Fine PEM (1997). The natural history of tuberculosis: the implications of age-dependent risks of disease and the role of reinfection. Epidemiol Infect.

[40] Cui Y, Shen H, Wang F, Wen H, Zeng Z, Wang Y, Yu C (2020). A long-term trend study of tuberculosis incidence in China, India and United States 1992–2017: a joinpoint and age–period–cohort analysis. Int J Environ Res Public Health.

[41] Wu P, Cowling BJ, Schooling CM, Wong IO, Johnston JM, Leung CC, Tam C-M, Leung GM (2008). Age–period–cohort analysis of tuberculosis notifications in Hong Kong from 1961 to 2005. Thorax.

[42] Strachan DP, Powell KJ, Thaker A, Millard FJ, Maxwell JD (1995). Vegetarian diet as a risk factor for tuberculosis in immigrant south London Asians. Thorax.

[43] Willis MD, Winston CA, Heilig CM, Cain KP, Walter ND, Mac Kenzie WR (2012). Seasonality of tuberculosis in the United States, 1993–2008. Clin Infect Dis.

[44] Stevenson CR, Forouhi NG, Roglic G, Williams BG, Lauer JA, Dye C, Unwin N (2007). Diabetes and tuberculosis: the impact of the diabetes epidemic on tuberculosis incidence. BMC Public Health.

[45] Kumar V, Singh A, Adhikary M, Daral S, Khokhar A, Singh S (2014). Seasonality of tuberculosis in Delhi, India: a time series analysis. Tuberc Res Treat.

[46] Lönnroth K, Williams BG, Stadlin S, Jaramillo E, Dye C (2008). Alcohol use as a risk factor for tuberculosis—a systematic review. BMC Public Health.

[47] Khatri GR, Frieden TR (2002). Controlling tuberculosis in India. N Engl J Med.

[48] Winston CA, Navin TR (2010). Birth cohort effect on latent tuberculosis infection prevalence, United States. BMC Infect Dis.

[49] Padmapriyadarsini C, Shobana M, Lakshmi M, Beena T, Swaminathan S (2016). Undernutrition & tuberculosis in India: situation analysis & the way forward. Indian J Med Res.

[50] Marimuthu P (2016). Tuberculosis prevalence and socio-economic differentials in the slums of four metropolitan cities of India. Indian J Tuberc.

[51] Prasad R, Suryakant RG, Singhal S, Dawar R, Agarwal GG (2009). A case-control study of tobacco smoking and tuberculosis in India. Ann Thorac Med.

