# Supplementary material for: Trends in incidence and mortality of tuberculosis in India over past three decades: a joinpoint and age–period–cohort analysis
Source: BMC Pulm Med. 2021 Nov 16;21:375. doi: 10.1186/s12890-021-01740-y (PMC8597252; doi:10.1186/s12890-021-01740-y)
Supplement: Supplementary file 1 — Additional File 1: Table 1. Sex-specific temporal trends in incidence and mortality of tuberculosis in India based on the joinpoint regression analysis (1990-2019). Table 2. Wald χ2 tests to test the significance of estimable functions. [file 12890_2021_1740_MOESM1_ESM.docx]

**Additional File 1: Table 1.** Sex-specific temporal trends in incidence and mortality of tuberculosis in India based on the joinpoint regression analysis (1990-2019).

| Factors | Trend 1 | | Trend 2 | | Trend 3 | | Trend 4 | | Trend 5 | | Trend 6 | | |  |
| --- | --- | --- | --- | --- | --- | --- | --- | --- | --- | --- | --- | --- | --- | --- |
|  | **Year** | **APC (95% CI)** | **Year** | **APC (95% CI)** | **Year** | **APC (95% CI)** | **Year** | **APC (95% CI)** | **Year** | **APC (95% CI)** | **Year** | **APC (95% CI)** | **AAPC (95% CI) (1990-2019)** | |
| Age Standardized Incidence rate | | | | | | | | | | | | | |  |
| Males | 1990-1994 | -2.38* (-2.68, -2.08) | 1994-2001 | 0.40* (0.23,0.56) | 2001-2007 | -1.92* (-2.13, -1.71) | 2007-2016 | -3.04* (-3.14, -2.94) | 2016-2019 | -0.45 (-0.93, 0.03) | - | - | -1.63* (-1.71, -1.54) | |
| Females | 1990-1994 | -3.13* (-3.43, -2.83) | 1994-2001 | -0.68* (-0.84, -0.51) | 2001-2017 | -2.95* (-2.99, -2.91) | 2017-2019 | 0.31 (-0.67, 1.30) | - | - | - | - | -2.21* (-2.29, -2.12) | |
| Both | 1990-1994 | -2.76* (-2.97, -2.55) | 1994-2001 | -0.11 (-0.23,0.01) | 2001-2006 | -2.41* (-2.62, -2.19) | 2006-2017 | -2.90* (-2.95, -2.85) | 2017-2019 | 0.43 (-0.26, 1.12) | - | - | -1.90* (-1.97, -1.83) | |
| Age Standardized Mortality rate | | | | | | | | | | | | | |  |
| Males | 1990-2001 | -2.80* (-3.30, -2.30) | 2001-2004 | -7.00 (-13.88, 0.43) | 2004-2019 | -4.02* (-4.34, -3.71) | - | - | - | - | - | - | -3.88* (-4.63, -3.11) | |
| Females | 1990-1992 | 1.59 (-4.23, 7.77) | 1992-1995 | -7.25* (-12.57,-1.62) | 1995-1999 | -1.70 (-4.56, 1.24) | 1999-2010 | -6.24* (-6.67, -5.81) | 2010-2019 | -3.47* (-3.99, -2.95) | - | - | -4.35* (-5.12, -3.57) | |
| Both | 1990-1992 | 0.90 (-4.82, 6.96) | 1992-1995 | -6.14* (-11.46, -0.50) | 1995-1999 | -1.64 (-4.46, 1.28) | 1999-2011 | -5.52* (-5.89, -5.15) | 2011-2014 | -1.85 (-7.41, 4.05) | 2014-2019 | -4.74* (-5.97, -3.48) | -4.11* (-5.03, -3.18) | |

APC, annual percent change; CI, confidence interval; AAPC, average annual percent change.

* Indicates the APC was significant different from zero at the alpha = 0.05 level

**Additional File 1: Table 2.** Wald χ2 tests to test the significance of estimable functions.

| Wald Test | Male Incidence | | | Female Incidence | | |
| --- | --- | --- | --- | --- | --- | --- |
|  | **Chi Square** | **df** | **P-Value** | **Chi Square** | **df** | **P-Value** |
| Net Drift = 0 | 1062.2713 | 1 | 0 | 935.6021 | 1 | 0 |
| All Age Deviations = 0 | 5408.1525 | 16 | 0 | 3234.5482 | 16 | 0 |
| All Period Deviations = 0 | 229.0609 | 4 | 0 | 31.7904 | 4 | 0 |
| All Cohort Deviations = 0 | 224.7357 | 21 | 0 | 471.6536 | 21 | 0 |
| All Period RR = 1 | 1279.7302 | 5 | 0 | 958.7686 | 5 | 0 |
| All Cohort RR = 1 | 1736.4418 | 22 | 0 | 2993.6141 | 22 | 0 |
| All Local Drifts = Net Drift | 224.5433 | 18 | 0 | 470.8659 | 18 | 0 |
|  | **Male Mortality** | | | **Female Mortality** | | |
| Net Drift = 0 | 3599.6666 | 1 | 0 | 3206.8239 | 1 | 0 |
| All Age Deviations = 0 | 887.9338 | 16 | 0 | 262.4942 | 16 | 0 |
| All Period Deviations = 0 | 40.0007 | 4 | 0 | 22.1667 | 4 | 0 |
| All Cohort Deviations = 0 | 114.3525 | 21 | 0 | 82.3314 | 21 | 0 |
| All Period RR = 1 | 3624.2958 | 5 | 0 | 3249.1842 | 5 | 0 |
| All Cohort RR = 1 | 5360.7968 | 22 | 0 | 3803.2087 | 22 | 0 |
| All Local Drifts = Net Drift | 112.1672 | 18 | 0 | 79.7909 | 18 | 0 |
